# Biomimetic synthesis of proline-derivative templated mesoporous silica for increasing the brain distribution of diazepam and improving the pharmacodynamics of nimesulide

**DOI:** 10.1080/10717544.2017.1359863

**Published:** 2017-08-01

**Authors:** Heran Li, Jianxin Wang, Jialiang Cong, Chen Wei, Jing Li, Hongzhuo Liu, Sanming Li, Mingshi Yang

**Affiliations:** a Wuya College of Innovation, Shenyang Pharmaceutical University, Shenyang, China;; b School of Pharmacy, Shenyang Pharmaceutical University, Shenyang, China;; c Faculty of Health and Medical Science, University of Copenhagen, Copenhagen, Denmark

**Keywords:** Mesoporous silica nanoparticle, brain targeting, biomimetic synthesis nanomaterial, drug delivery

## Abstract

Herein a new kind of proline-derivative templated mesoporous silica with curved channels (CMS) was biomimetically synthesized and applied as carrier to improve the drug dissolution and bioavailability of hydrophobic diazepam (DZP) and nimesulide (NMS). Drugs can be incorporated into CMS with high efficiency; during this process, they successfully transformed to amorphous phase. As a result, the dissolution rate of DZP and NMS was significantly improved. Biodistribution study confirmed that CMS converted DZP distribution in mice with the tendency of lung targeting and brain targeting. At 45 min postadministration, the concentrations of DZP in plasma, lung and brain were 8.57-fold, 124.94-fold and 19.55-fold higher from 1:3 DZP/CMS sample than that of pure DZP sample, respectively. At 90 min postadministration, the content of DZP in brain was 62.31-fold higher for 1:3 DZP/CMS sample than that of pure DZP. Besides, the anti-inflammatory and analgesic effects of 1:3 NMS/CMS were systematic evaluated using mouse ankle swelling test (MAST), mouse ear swelling test (MEST) and mouse writhing test (MWT). The results indicated that after incorporating into CMS, the therapeutic effects of NMS were obviously improved, and the inhibition rates of 1:3 NMS/CMS in all pharmacodynamics tests varied from 102.2% to 904.3%.

## 1. Introduction

In the area of clinical work, the low solubility of the drugs causes a series of problems, such as low bioavailability, insufficient clinical outcomes and dose escalation due to the poor absorption in the gastrointestinal tract after oral administration (Dolinina et al., 2016; Maleki & Hamidi, 2016). However, nearly 2/5 of the marketed immediate-release oral drugs are practically insoluble, including diazepam and nimesulide (Zhang et al., 2015). Diazepam is a positive allosteric modulator of the GABA type A receptors belonging to the benzodiazepine family which is very slightly soluble in water, and is commonly used for the treatment of anxiety, muscle spasms, seizures, etc. (Riss et al., 2008; Giacomini et al., 2016). Nimesulide is a kind of non-steroidal anti-inflammatory drug (NSAID) which can be classified as BCS II (Kress et al., 2016). It works by blocking the production of prostaglandins, inhibiting COX-2 selectively, and is developed for the treatment of acute pain (short term), and primary dysmenorrhea (period pains) (Luo et al., 2015). In these cases, improving the solubility and controlling the drug release behavior are the core strategies to increase the oral bioavailability of poorly water-soluble drugs. Many efforts have been made to overcome the low bioavailability of the poorly water-soluble drugs, such as co-solvents (Jogunola et al., 2016), complexation (Zhang et al., 2017), salification (Fini et al., 2012), micronization (Fu et al., 2013), solid dispersion (Maher et al., 2016), vesicle (Yan et al., 2016), micelle (Liu et al., 2017), cyclodextrin (Loftsson & Brewster, 2012), etc. (Meng et al., 2017). With the development of technology, materials were developed into nanoscale level and were found applications in many fields including drug delivery, catalysis, sensors, adsorption and separation (Goscianska et al., 2015; Li et al., 2015c). Since the M41S family was first discovered in the early 1990s, mesoporous silica has attracted increasing interest because of their unique properties such as relatively large surface area and pore volume, excellent pore structure and rich morphology, uniform and adjustable size and *in vivo* biosafety, and has been proved to be promising candidates for biomedical applications and drug delivery (Putz et al., 2015; Wang et al., 2015; Guha et al., 2016). Compared with traditional methods, the silica-based mesoporous materials have additional advantages to be considered as a novel drug carrier. Firstly, the well-developed nanometer-sized pores effectively limit the crystallization of drug and keep the drug in an amorphous state to increase the solubility of drug in water (Hu et al., 2015). Secondly, the structural features of mesoporous silica nanoparticles provide hydrophobic microenvironment which enhances the stability of drugs and is well suited for drug loading (Mekaru et al., 2015). Thirdly, the excellent biological compatibility and safety of silicon materials enable their application as novel drug delivery systems (Xie et al., 2016).

Intricate nanostructure and nanopatterning with precise control usually appeared in diatoms and sponges (Naik et al., 2003; Ford & Yang, 2007). Biominerals often show hierarchical organization ranging from nanoscale to microscale (Jin & Yuan, 2006). During the biomineralization process, organisms are formed within their cell walls and reproduced generation after generation (Zhao & Su, 2012). Biomimetic synthesis of mesoporous silica has become one of the current topics in silica materials since biomineralization is a bottom-up process occurred at ambient condition and is genetically controlled by specialized biomacromolecules including polysaccharides, polypeptides and amino acid derivatives (Naik et al., 2003; Jin & Yuan, 2006). For example, controlled formation of arched and elongated biosilica structures was synthesized by Rajesh et al., using a synthetic peptide (Naik et al., 2003). Folate-templated mesoporous silica was obtained by using folate supramolecular templates and it grew followed a standard nucleation and growth mechanism (Atluri et al., 2013). Besides, aspartic acid block copolymer-imprinted mesoporous silica which can be used for chiral separation was successfully obtained by Paik et al., by using amphiphilic molecules composed of d/l-aspartic acid and PEG (Paik et al., 2010). A novel nanoporous silica xerogel was prepared using polyethyleneimine with biomimetic method and used as drug carrier to investigate the controlled release behavior of this material (Li et al., 2014).

In the present study, mesoporous silica nanosphere with curved channels was biomimetically synthesized based on solgel method by using synthesized surfactant *N*-palmitoyl-l-proline as template, 3-aminopropyltriethoxysilane (APTES) as CSDA and tetraethoxysilane (TEOS) as the main inorganic source. The morphology and structural features of CMS were investigated using transmission electron microscopy (TEM), scanning electron microscopy (SEM), X-ray diffraction (XRD) and nitrogen adsorption/desorption measurement. Diazepam (DZP) and nimesulide (NMS) were selected as model drugs, and they were loaded into CMS according to the solvent deposition method. The drug loading and release behaviors of model drugs with CMS as carries were deeply studied to explore the controlled release functions of CMS. The drug crystalline state was systemically characterized using X-ray diffraction (XRD), differential scanning calorimetry (DSC), thermal gravity analysis (TGA) and Fourier transform infrared spectrometer (FTIR). Biodistribution study of samples after oral administration was performed to confirm the *in vivo* behavior of CMS. Besides, the anti-inflammatory effect of NMS/CMS was systematically evaluated. In particular, the biodistribution and pharmacodynamic studies of drug/CMS samples were compared with that of the samples composed of drugs and the commercially available silica-based mesoporous material MCM41.

## 2. Experimental

### 2.1. Materials

1-(3-dimethylaminopropyl)-3-ethylcarbodiimide hydrochloride (EDCI), 1-hydroxybenzotriazole (HOBT), dimethylformamide (DMF) and 4-(dicyanomethylene)-2-methyl-6-(4-dimethylaminostyryl)-4H-pyran (DCM) were purchased from Shanghai Jinjinle Industrial Co., Ltd. (Shanghai, China). 3-aminopropyltriethoxysilane (APTES) and λ-carrageenan were purchased from Chengdu Xiya Chemical Technology Co., Ltd. (Chengdu, China). Palmitic acid, l-proline methyl ester and tetraethoxysilane (TEOS) were purchased from Yangzhou Baosheng Biochemical Co., Ltd. (Yangzhou, China). MCM41 (average pore diameter: 3–5 nm; particle size: 200–300 nm) was obtained from Suzhou Jiedi Nano Technology Co., Ltd., (Suzhou, China). Diazepam and carbamazepine were produced by Zhengzhou Ancheng Biological Technology Co., Ltd. (Zhengzhou, China). Nimesulide was obtained from Wuhan Dinghui Chemical Co., Ltd. (Wuhan, China). Double distilled water was prepared by ion exchange and used in all experiments.

### 2.2. Synthesis of *N*-palmitoyl-l-proline

In a typical run, 0.33 g l-proline methyl ester hydrochloride was dissolved in 10 ml TMF, to which 1 ml triethylamine was added dropwise under stirring. After 20 min, palmitic acid (0.51 g), EDCI (0.46 g) and HOBT (0.23 g) were added and stirred for another 20 min. Then, 2 ml DCM was introduced to the system and the mixture was transferred to an oil bath at 60 °C, and the reaction was supervised by thin layer chromatography (TLC) until brown liquid was obtained. Then, the mixture was evaporated to remove the organic phase and carefully washed by a saturated aqueous solution of NaHCO_3_ and water. After precipitation, the crude obtained was filtered, dried, crushed and weighted without recrystallization (because of the low melting point).

*N*-palmitoyl-l-proline was prepared by hydrolyzing of *N*-palmitoyl-l-proline methyl ester. In detail, *N*-palmitoyl-l-proline methyl ester was dissolved in 100 ml mixture solution composed of 70% ethyl alcohol, 30% water (v/v) and 12.39 g NaOH under stirring for about 6 h. Then, the *N*-palmitoyl-l-proline obtained was separated from organic solvent using reduced pressure distillation, adjusted pH to 2–3 by HCl, filtered, washed, dried and weighted.

### 2.3. Preparation of CMS

CMS was synthesized by a procedure established by our laboratory using APTES as CSDA and TEOS as silica source. Typically, 0.43 g of *N*-palmitoyl-l-proline was dispersed in 10 ml water at 80 °C under stirring. Afterwards, 10 ml NaOH was added dropwise and the system was cooled to 25 °C. After stirring for at least 1 h, a mixture of APTES (0.24 ml) and TEOS (1.57 ml) was introduced to the solution, and the system was stirred until milky white turbid solution was obtained. Then, the mixture was left at ambient condition statically for 24 h. After that, the precipitation was separated by centrifuge, washed several times with ethanol and deionized water, dried, grinded and finally calcined at 550 °C for 6 h with a slow heating rate.

### 2.4. Drug loading process

The solvent deposition method was carried out to load diazepam (DZP) and nimesulide (NMS) into CMS, respectively. In detail, DZP was first dissolved in acetone to get a high-concentration solution (10 mg/ml). DZP loaded to CMS was achieved by adding certain amount of CMS into the solution of DZP at a series of drug/carrier ratios (2:1, 1:1, 1:2 and 1:3, w/w). Then, the system was sealed and left under ambient condition for at least 24 h with gentle stirring. After that, the mixture was washed with methanol to remove the drugs adsorbed on the surface of CMS and dried under vacuum. The products were named as 2:1 DZP/CMS, 1:1 DZP/CMS, 1:2 DZP/CMS and 1:3 DZP/CMS, respectively. The drug loading contents of DZP/CMS samples were determined by taking 5 mg of DZP/CMS samples and extracting DZP completely using ethanol under the treatment of ultrasound. Finally the concentration of DZP was measured by ultraviolet (UV) spectroscopy (UV-1750, Shimadzu, Japan) at a wavelength of 240 nm.

NMS/CMS samples were prepared by the same method and denoted as 2:1 NMS/CMS, 1:1 NMS/CMS, 1:2 NMS/CMS and 1:3 NMS/CMS, respectively. In particular, the NMS loading process should be operated in the darkroom. Drug loading content was measured by taking 5 mg of NMS/CMS samples and ultrasonically extracting the loaded NMS completely using NaOH (0.05 mol/L). Finally, the content of NMS loaded was analyzed by UV spectroscopy at the wavelength of 393 nm.

DZP/MCM41 samples and NMS/MCM41 samples were also prepared by solvent evaporation method at the drug/carrier ratio of 1:3 (w/w) and were referred to as 1:3 DZP/MCM41 and 1:3 NMS/MCM41, respectively.

### 2.5. Characterizations

TEM images and SEM images of the prepared CMS were obtained using a TEM instrument (Tecnai G2-F30, FEI, the Netherlands) and a SEM instrument (JSM-6510 A, JEOL, Japan), respectively. The nitrogen adsorption and desorption analysis was carried out using an adsorption analyzer (Vsob-2800 P, China) at −196 °C to get the information of the surface area and pore volume of CMS. The specific surface area (S_BET_) was estimated using the Brunauer–Emmett–Teller (BET) method. Barrett–Joyner–Halenda (BJH) method was used to calculate the pore size distribution (PSD) from the corresponding desorption branch of the ad/desorption isotherm. The total pore volume (V_t_) was derived from N_2_ adsorption at the relative pressure of 0.99. FTIR study (Spectrum 1000, PerkinElmer, Waltham, MA) and *in situ* FT-IR study (IR Tracer-100, Shimadzu, Japan) were carried out to obtain the structural information and the interaction between drug and carrier. Prior to the study, samples were carefully mixed with dried KBr and crushed to prepare KBr disks. XRD patterns of samples were acquired using an X-ray diffractometer (EMPYREAN, PANalytical B.V., the Netherlands) at 30 mA and 30 kV with a Ni-filtered CuKa line as the source of radiation. DSC curves were generated on a differential scanning calorimeter (DSC, Q1000, TA Instrument, New Castle, DE). Each sample was heated from 20 to 200 °C with a heating rate of 5 °C/min under a nitrogen flow. TGA analysis was performed on DTA-TG instrument (DTG-60, Shimadzu, Japan) from 50 to 550 °C at a heating rate of 10 °C/min.

### 2.6. *In vitro* release behavior

Dissolution studies of samples were carried out according to a USP II paddle method at 37 °C with a stirrer rotation speed of 50 rpm in a dissolution apparatus (ZRD6-B, Shanghai Huanghai Medicament Test Instrument Factory, Shanghai, China). Pure DZP (4 mg) and DZP/CMS samples (containing 4 mg DZP) were, respectively, exposed to 250 ml of deionized water. At predetermined time intervals, 5 ml samples were taken out and replaced by fresh medium immediately. Then, samples were passed through a 0.22 μm membrane filter and were transferred to UV/Vis analysis at the wavelength of 240 nm. Besides, the dissolution of DZP and 1:3 DZP/CMS was also investigated in the medium of simulated gastric fluid (SGF, pH 1.0) and simulated intestinal fluid (SIF pH 6.8) to further evaluate the *in vitro* release behavior of samples.

Samples containing 5 mg NMS were put into 250 ml phosphate buffer solutions (PBS, pH 7.8) at 37 °C. Taking, processing and analyzing of samples were the same as the dissolution studies of DZP and the absorbance was measured at 393 nm.

### 2.7. Animals

All animal experiments were approved by the Shenyang Pharmaceutical University Animal Care and Use Committee (Liaoning, China). Animals received care in compliance with the guidelines for the Care and Use of Laboratory Animals.

### 2.8. Biodistribution of diazepam

#### 2.8.1 Experimental design

The biodistribution of the test silica formulation (1:3 DZP/CMS) was compared with that of the pure DZP and the drug loading samples composed of DZP and commercially available silica-based carrier MCM41. 24 male SPF-grade Kunming mice weighing 18–22 g were randomly assigned into three groups: DZP group, 1:3 DZP/CMS group and 1:3 DZP/MCM41 group. Before the experiment, animals were fasted overnight with free access to water. Samples equivalent to 1 mg DZP were suspended in normal saline and were provided to mice by intragastric administration. At 45 min and 90 min after oral administration, blood samples were taken by removing eyeball. Then, animals were sacrificed, and major organs including the brain, heart, liver, kidney, spleen and lungs were rapidly collected, weighed, disrupted in a manual homogenate instrument, extracted with cold physiological saline, centrifugated twice at 6000 rpm for 8 min to get supernatant and frozen at −20 °C. The content of DZP in blood and organs was measured by high-performance liquid chromatography (HPLC).

#### 2.8.2. Sample preparation and HPLC analysis of DZP

Firstly, 20 μl of internal standard solution (carbamazepine, 20 μg/ml) and 200 μl of alkaline liquid (NaOH, 0.5 mol/L) were added to 200 μl of biological samples and mixed by vortex mixer (3 min). Then, 2.6 ml of extraction solvent (diethyl ether) was introduced and the mixture was further vortexed for 5 min. After centrifugation at 5000 rpm for 10 min, the organic phase (2 ml) was carefully removed and evaporated to dryness at 40 °C under a gentle stream of N_2_. Then, the dried residue was reconstituted with 100 μL mobile phase, vortexed, centrifuged, and delivered to HPLC analysis (20 μl).

The HPLC method was established to measure the concentrations of DZP in blood and organs. The HPLC system was equipped with a Hitachi 10-AT pump and a UV detector which was set at 254 nm. The separation was carried out on a Thermo ODS-2 Hypersil C18 column (250 mm × 4.6 mm, 5 mm, Waltham, MA) preceded by a JanuSep C18 pre-column (Benxi, China). The flow rate was kept at 1 ml/min with a mobile phase consisted of methanol and water (75:25, v/v). The column temperature was maintained at 25 °C. The DZP concentrations were calculated by internal standard method.

### 2.9. Pharmacodynamic studies of NMS

#### 2.9.1. Mouse ankle swelling test

A swelling inhibition study comprised of 18 male Sprague Dawley rats weighted 180–220 g was carried out to compare the anti-inflammatory profile of 1:3 NMS/CMS (NMS/CMS middle-dose group) vs. NMS/MCM41 (1:3 NMS/MCM41 group). Normal saline (NS group) and pure NMS (NMS group) were given to rats and served as negative control and positive control, respectively. Rats in NS group were administered orally of normal saline only, and rats in other groups were administered orally at a dose equivalent to 25 mg/kg NMS. To evaluate the effect of dosage, another two groups of rats were exposed to half and twice of the dose in NMS/CMS middle-dose group which equaled to 12.5 mg/kg and 50 mg/kg of NMS and denoted as the NMS/CMS low-dose group and the NMS/CMS high-dose group, respectively. At 30 min postadministration, 0.1 ml λ-carrageenan (1%, w/v) was injected to the right ankle area of each rat to induce acute inflammatory models. Then, the circumference of right ankle was measured at 0.5 h, 1 h, 1.5 h, 2 h, 3 h and 4 h after injection of λ-carrageenan. It should be noticed that, before the test, the initial circumference of right ankle of each rat was measured and the swelling degree was evaluated by the variation of ankle circumference. The repression rate and the swelling rate of the ankle swelling were calculated using the following Equations (1) and (2), respectively:

(1) $$Repression\;rate\;(\%)=\frac{{\left(c_{t}-c_{0}\right)}_{negative\;control}-{\left(c_{t}-c_{0}\right)}_{test}}{{\left(c_{t}-c_{0}\right)}_{negative\;control}-{\left(c_{t}-c_{0}\right)}_{positive\;control}}\times 100$$

(2) $$Swelling\;rate\;(\%)=\frac{c_{t}-c_{0}}{c_{0}}\times 100$$

where c_0_ is the initial circumference and c_*t*_ is the circumference measured at different time points.

#### 2.9.2. Mouse ear swelling test

The mouse ear swelling test was developed to further evaluate the swelling inhibition potential of NMS/CMS. In detail, 16 male Kunming mice weighing 18–22 g were randomly divided into four groups (*n* = 4): NS group (negative control group), NMS group (positive control group), NMS/CMS group (1:3 NMS/CMS) and NMS/MCM41 group (1:3 NMS/CMS). Samples containing 0.6 mg NMS were suspended in normal saline and imposed to mice in all groups by intragastric administration. Mice in NS group were exposed to normal saline only. At 30 min after administration, 0.1 ml xylene was applied on each side of the left ear of mice, and the right ear regarded as a reference was left untreated. After 50 min, mice were executed, both ears were removed, and tissues with diameter of 0.6 cm were cut off by hole punch. The weight of the tissues cut from left and right ears was measured by electronic scale. The repression rate and the degree of ear swelling were, respectively, expressed as:

(3) $$Repression\;rate\;(\%)=\frac{{\left(w_{L}-w_{R}\right)}_{negative\;control}-{\left(w_{L}-w_{R}\right)}_{test}}{{\left(w_{L}-w_{R}\right)}_{negative\;control}-{\left(w_{L}-w_{R}\right)}_{positive\;control}}\times 100$$

(4) $$Swelling\;rate\;(\%)=\frac{w_{L}-w_{R}}{w_{R}}\times 100$$

where w_*L*_ is the weight of tissue cut from the left ear and w_*R*_ is the weight of tissue cut from the right ear of the same mice.

#### 2.9.3. Mouse writhing test

The antinociceptive effects of NMS/CMS and NMS/MCM41 were investigated and compared in mouse writhing test. 16 mice were randomly assigned into four groups (*n* = 4): NS group (negative control group), NMS group (positive control group), NMS/CMS group (1:3 NMS/CMS) and NMS/MCM41 group (1:3 NMS/MCM41). Mice were orally administered with the samples which were equivalent to 0.6 mg NMS. At 1 h postadministration, mice were intraperitoneally injected with 0.4 ml acetic acid 0.4% (v/v) to induce writhing and were individually housed in a mouse cage (20 × 30 × 12 cm). Afterwards, the number of writhes (a writhe was account as abdominal constriction or stretching of at least one hind limb) for each mice was recorded within 20 min postinjection. The repression rate of the test formulations was expressed as:

(5) $$Repression\;rate\;(\%)=\frac{n_{negative\;control}-n_{test}}{n_{negative\;control}-n_{positive\;control}}\times 100$$

where *n* is the number of writhes recorded from each mice.

### 2.10. Statistical analysis

All *in vitro* studies were performed in triplicate and the data were expressed as mean ± SD. The *in vivo* results are expressed as the means ± SD. Statistical analysis was analyzed by Student’s *t*-test or one-way analysis of variance followed by Dunnet’s *t*-test to identify levels of significance between the groups. Statistical significance was set as *p* < .05.

## 3. Results and discussion

### 3.1. Formation mechanism of CMS

**Figure 1.**
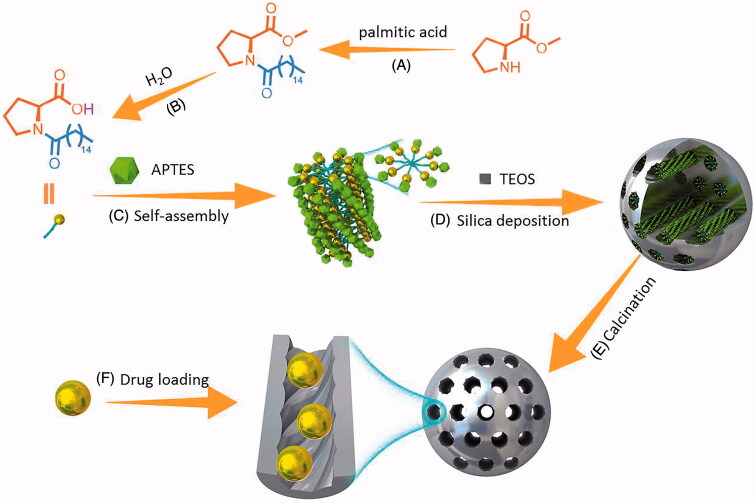
Schematic illustration of synthesize routes of *N*-palmitoyl-l-proline (A,B) and CMS (C–E). (A) Dehydration–condensation reaction catalyzed by EDCI and HOBT; (B) ester hydrolysis reaction; (C) self-assembly process; (D) silica deposition procedure; (E) removal of temples by calcination; (F) drug loading process.

CMS was successfully synthesized upon a co-structure directing agent (CSDA) method through the solgel reaction. In the present work, *N*-palmitoyl-l-proline was served as template, TEOS was used as the main silica source and APTES was employed as structure directing agent. As described in Figure 1, during the synthesis process, alkoxysilane sites of APTES polymerized with TEOS to form framework; meanwhile, positively charged ammonium sites of APTES electrostatically interact with negatively charged part of the *N*-palmitoyl-l-proline (Li et al., 2015b). The curved mesostructure was initiated by the dynamic self-assembly of *N*-palmitoyl-l-proline and imprinted along with the process of silica deposition. It should be mentioned that, during the synthesis process, amines actively catalyzed the condensation of silica precursors; thus, the synthesis can be classified to biomimetic synthesis (Naik et al., 2003; Li et al., 2015b).

### 3.2. Characterization of CMS

**Figure 2.**
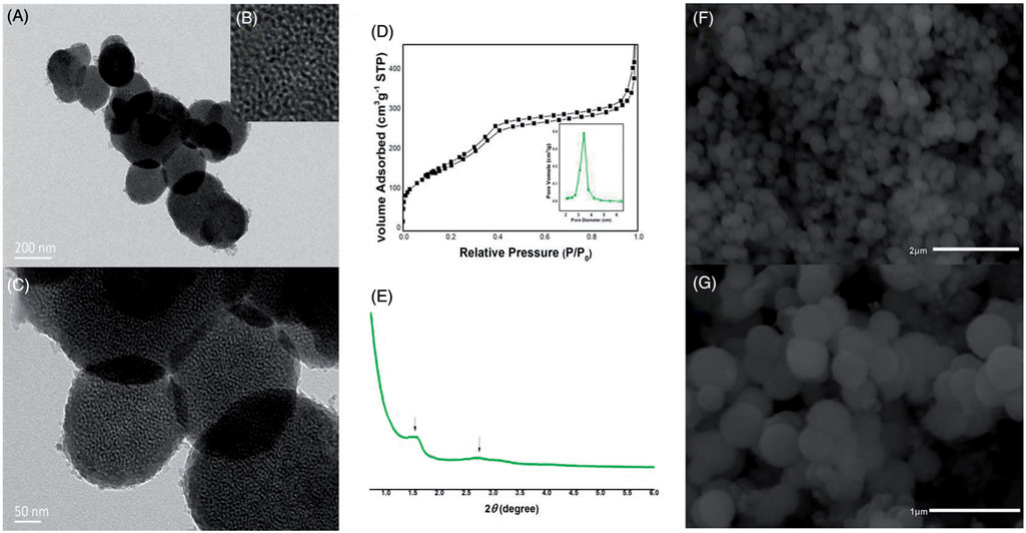
(A–C) TEM images of CMS, (B) is the local structure of (A); (C) is the amplifying image of (A); (D) nitrogen adsorption/desorption isotherm and pore size distribution curve of CMS; (E) SAXD pattern of template-free CMS; (F–G) SEM images of CMS.

**Figure 3.**
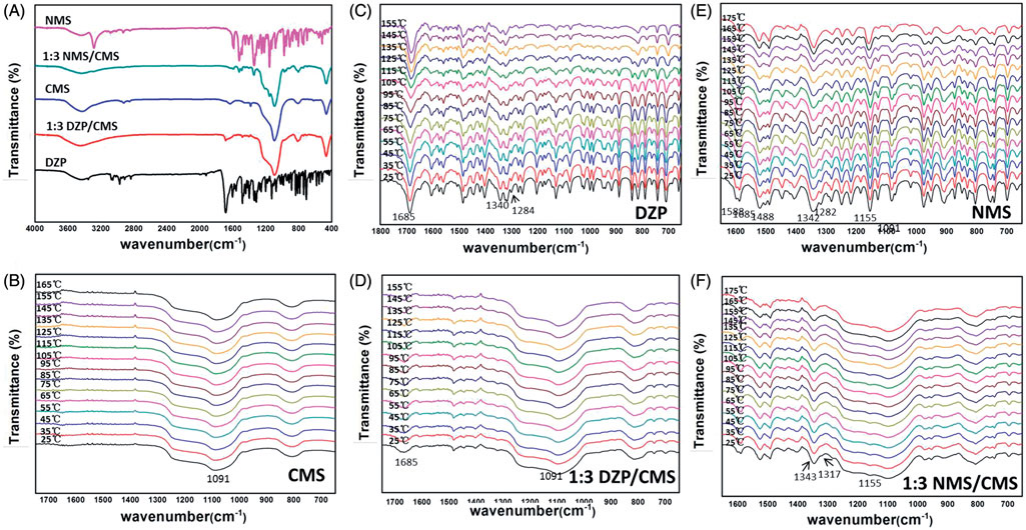
FTIR spectra of samples (A), and *in situ* FTIR spectra of CMS (B), DZP (C), 1:3 DZP/CMS (D), NMS (E) and 1:3 NMS/CMS (F).

**Figure 4.**
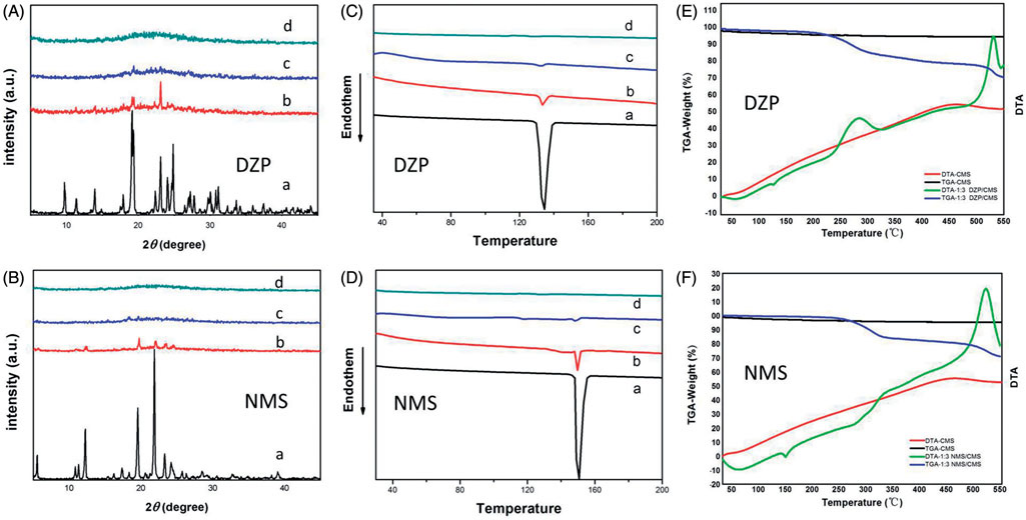
(A) XRD patterns of (A) DZP; (B) physical mixture of DZP/CMS (1:3, w/w); (C) 1:3 DZP/CMS; (D) CMS; (B) XRD patterns of (A) NMS; (B) physical mixture of NMS/CMS (1:3, w/w); (C) 1:3 NMS/CMS; (D) CMS; (C) DSC thermograms of (A) DZP; (B) physical mixture of DZP/CMS (1:3, w/w); (C) 1:3 DZP/CMS; (D) CMS; (D) DSC thermograms of (A), NMS; (B) physical mixture of NMS/CMS (1:3, w/w); (C) 1:3 NMS/CMS; (D) CMS; (E) TGA-DTA thermograms of DZP, 1:3 DZP/CMS and CMS; (F) TGA-DTA thermograms of NMS, 1:3 NMS/CMS and CMS.

As can be seen in Figure 2, CMS particles were nanospheres with shell–core structure and were about 200–300 nm in diameter. A large number of disordered curved channels were clearly visible with wormhole arrangement. Figure 2(D) showed the nitrogen adsorption–desorption isotherm and pore size distribution curve of CMS. It can be seen that CMS exhibited a type IV feature with a capillary condensation step, indicating the presence of mesopores (Nakanishi et al., 2014; Li et al., 2016). The average pore size estimated by BJH method was 3.42 nm, the BET surface area of CMS was 568.27 m^2^/g, and the total pore volume was found to be 0.75 cm^3^/g. In the FTIR spectrogram of CMS (Figure 3(A)), Si–O–Si bending vibration at 466.1 cm^−1^ and Si–O–Si antisymmetric stretching vibration at 1096.1 cm^−1^ can be detected, indicating the successful synthesis of silica materials (Nakanishi et al., 2014; Finlay et al., 2015; Li et al., 2015a). Moreover, the XRD (Figures 2(E), 4(A)) and DSC patterns (Figure 4(C,D)) indicated that CMS was a kind of amorphous material with mesoporous structure (Finlay et al., 2015; Hashemikia et al., 2016).

### 3.3. Drug loading content

As summarized in Table 1, the drug loading content of CMS was very high, and only a small proportion of drug was lost during the loading process. The loading content of DZP and NMS into CMS was decreased with the increasing proportion of CMS. For both drugs, the drug loading capacity of CMS (26.69% for 1:3 DZP/CMS and 23.06% for 1:3 NMS/CMS) was higher than the drug loading capacity of MCM41 (21.17% for 1:3 DZP/MCM41 and 20.57% for 1:3 NMS/MCM41). The results suggested stronger interactions between drugs and CMS, indicating that CMS could load drugs with high efficiency, which was an important feature to be extensively used as carrier of sustained-release and controlled-release preparation.

**Table 1.** Drug loading capacity (%) of samples.

Sample	Drug loading capacity (%)	Sample	Drug loading capacity (%)
2:1 DZP/CMS	57.31 ± 1.07	2:1 NMS/CMS	63.63 ± 1.56
1:1 DZP/CMS	49.80 ± 2.14	1:1 NMS/CMS	49.36 ± 1.28
1:3 DZP/CMS	33.45 ± 0.87	1:2 NMS/CMS	31.55 ± 0.27
1:3 DZP/CMS	26.69 ± 0.92	1:3 NMS/CMS	21.17 ± 0.94
1:3 DZP/MCM41	23.06 ± 1.78	1:3 NMS/MCM41	20.57 ± 1.22

### 3.4. Physical state of drug-loaded samples

#### 3.4.1. FTIR

Structural information of drug-loaded samples was illustrated by FTIR. As shown in Figure 3(A), DZP showed aromatic hydrocarbon peak at 3055 cm^−1^, which corresponded to C–H stretching vibration of benzene ring, and peaks at 2911 cm^−1^ and 1483 cm^−1^ belonging to the C–H stretching vibration and N–H bending vibration of the –CH_3_ groups, respectively (Mielcarek et al., 2011; Yamamoto et al., 2016; Bédard et al., 2017). After loading into CMS, most of the characteristic peaks of DZP disappeared, suggesting that DZP was successfully incorporated into CMS. Meanwhile, hydrogen bonding could be formed between the silanol groups of CMS and the carbonyl functions of DZP, which resulted in a red shift and a peak broadening of the peak at 1685 cm^−1^ relative to the C = O group (Mielcarek et al., 2011).

More details are announced in the *in situ* FITR spectra of DZP and 1:3 DZP/CMS. As can be seen from Figure 3(C), the intensity of the main characteristic peaks of DZP decreased gradually with the increasing temperature. Furthermore, peaks in the region of 1100–900 cm^−1^ disappeared gradually. Peaks at 1340 cm^−1^ and 1323 cm^−1^, which were assigned to in-plane C–H bending vibration of the benzene ring, had shifted to the lower wavenumbers, indicating the π–π interactions between benzene rings (Moros et al., 2007). Both the spectra of DZP and 1:3 DZP/CMS (Figure 3(D)) showed redshift of the wavenumber of carbonyl group peak from 1685 to 1681 cm^−1^, and from 1683 to 1678 cm^−1^, respectively. However, the intensity of peaks *in situ* FTIR spectra of 1:3 DZP/CMS changed slightly with temperature.

FTIR spectra of NMS before and after drug loading were also investigated and were shown in Figure 3(A). The characteristic peaks of the aromatic secondary amine can be observed, including the peak at 3283 cm^−1^ that attributed to the N–H stretching vibration and the peak at 1317 cm^−1^ belonging to the stretching vibration of C = N (de Paiva et al., 2012). However, in the spectra of 1:3 NMS/CMS, the peaks of benzene ring and secondary amine decreased significantly. It seemed that, NMS was successfully entered into the curved mesoscopic channels of CMS and hydrogen bonds were formed between the secondary amine groups of NMS and silanol groups of CMS during the drug loading process (Ramos & Diogo, 2016). The *in situ* FTIR spectra of NMS (Figure 3(E)) showed blueshift of the wavenumber of sulfonyl group characteristic peaks from 1155 to 1159 cm^−1^, which was related to the weakening of the S = O bond. Besides, the redshifts of the absorption bands from 1342 to 1340 cm^−1^ and from 1282 to 1280 cm^−1^ with decrease of intensity were, respectively, identified as the stretching of NO_2_ group and C–N group, indicating the consumption of intermolecular interaction (Huerta et al., 2015; Ramos & Diogo, 2016). However, in comparison with NMS, peaks belonging to the NO_2_ group and C–N group in the *in situ* spectra of 1:3 NMS/CMS (Figure 3(F)) showed no shift of wavenumber during the heating process, indicating a more stable environment.

#### 3.4.2. XRD and DSC

The physical states of DZP and NMS before and after drug loading process were determined by XRD and DSC, respectively. As shown in Figure 4(A), CMS comprised a broad band, characterized as amorphous single-phase material. The diffraction patterns of pure DZP and pure NMS were highly crystalline in nature as indicated by the numerous peaks. However, after drug loading process, the diffraction peak of 1:3 DZP/CMS sample showed amorphous broad peak, suggesting that DZP was loaded into CMS in amorphous state. Similar situation was found in the case of 1:3 NMS/CMS sample, and no crystalline NMS diffraction peak was detected. It seemed that the twisty mesopores of CMS and the limited living space of drugs prevented the crystallization of drugs, which were evidenced by the absence of crystalline peaks (Verheyen et al., 2001; Singh et al., 2015).

The DSC trace conducted on CMS was almost a smooth line with no melting point depression (Figure 4(C)). The DSC thermograms of DZP and NMS possessed single melting peak at 149.5 °C (Figure 4(C)) and 134.5 °C (Figure 4(D)), respectively (Nunes et al., 2016; Yamamoto et al., 2016; Bédard et al., 2017). The melting peaks of crystallographic DZP and NMS in the physical mixture of DZP/CMS (1:3, w/w) and NMS/CMS (1:3, w/w) can be easily detected with reductions of peak intensity. However, the DSC curves of 1:3 DZP/CMS and 1:3 NMS/CMS showed no melting peak for drugs, suggesting that DZP and NMS were existed in amorphous state. It should be noticed that DSC results were in agreement with XRD findings. Therefore, based on XRD and DSC results, after loading DZP or NMS into CMS, crystal form of drugs had been successfully converted to amorphous state.

Figure 4(E,F) showed the TGA and DTA curves of 1:3 DZP/CMS, 1:3 NMS/CMS and CMS, respectively. The DTA results of 1:3 DZP/CMS and 1:3 NMS/CMS were inconsistent with the DSC results when the temperature was lower than 200 °C, and NMS began to decompose when the temperature was higher than 450 °C. Besides, 1:3 DZP/CMS sample had undergone a two-stage decomposition at 270 °C and 500 °C, respectively. Meanwhile, the TGA curve of CMS was almost a horizontal line with a slightly weight loss (ca. 4.67%), which was related to the water adsorbed into CMS. As a result of the adsorbed water, the weight loss of 1:3 DZP/CMS (ca. 68.69%) and 1:3 NMS/CMS (ca. 71.09%) was also a little higher than the drug loading content measured before.

### 3.5. *In vitro* drug release study

**Figure 5.**
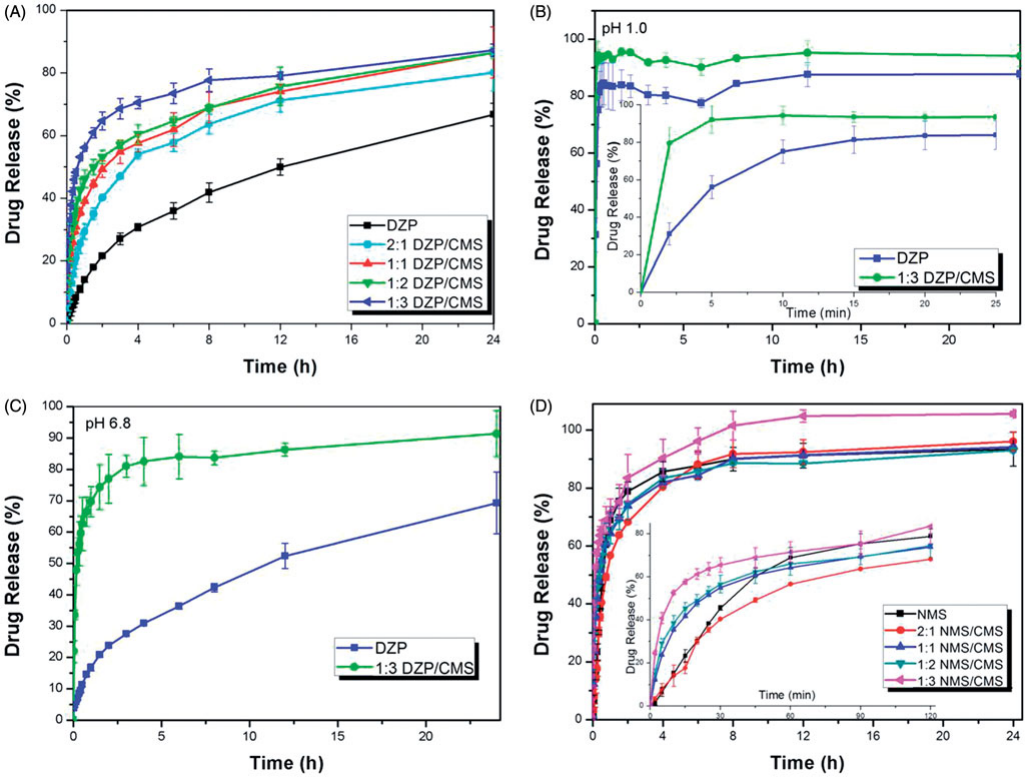
*In vitro* release profiles of samples. (A) The release profiles of DZP, 2:1 DZP/CMS, 1:1 DZP/CMS, 1:2 DZP/CMS and 1:3 DZP/CMS in water; (B) the release profiles of DZP and 1:3 DZP/CMS in SGF; (C) the release profiles of DZP and 1:3 DZP/CMS in SIF; (D) the release profiles of NMS, 2:1 NMS/CMS, 1:1 NMS/CMS, 1:2 NMS/CMS and 1:3 NMS/CMS in PBS.

A series of DZP/CMS samples were prepared, the release behaviors of them were studied, and the release profiles were presented in Figure 5(A). The result confirmed that DZP/CMS samples released much faster than the pure DZP. Their cumulative release percentage was more than 80% within 24 h, compared to that of pure DZP which released only 66.75%. Among them, best result came out from 1:3 DZP/CMS sample, which could release 87.25% of drug within 24 h. It should be noted that DZP release rate was determined by the drug/carrier weight ratio, and it increased with the increasing CMS proportion due to the inhibition of crystallization. In detail, the limitation of living space and the hydrophilic microenvironment of the carrier allowed the amorphization of the drug, which observably increased the solubility of poorly water-soluble drugs as a result of the higher energy state (Li et al., 2015a). On the other hand, the crystal growth of drug can be restricted by the very porous nature of CMS, which enhanced the dissolution rate of drug (Nakanishi et al., 2014).

To further study the influence of pH on the dissolution behavior of 1:3 DZP/CMS sample, *in vitro* drug release study was performed in SGF medium (pH 1) and SIF medium (pH 6.8), respectively. As can be seen in Figure 5(B,C), the dissolution rate of DZP can be obviously improved after loading into CMS in both SGF and SIF. However, it is worth mentioning that the drug release rate of 1:3 DZP/CMS in SGF was a little faster than that in SIF and in water with a burst release, and the final release ratio was higher than 95%. Besides, pure DZP also presented faster release in SGF rather than SIF and water (87.73% in SGF, 69.32% in SIF and 66.75% in water). The difference of release behavior may be attributed to the difference of saturated solubility of DZP in different dissolution media, which was 6.6 mg ml^−1^ in SGF, 0.046 mg ml^−1^ in SIF and 0.054 mg ml^−1^ in water (Aman-Pommier & Degobert, 2015).

The dissolution profiles of pure NMS, 2:1 NMS/CMS, 1:1 NMS/CMS, 1:2 NMS/CMS and 1:3 NMS/CMS were also investigated, and the results were presented in Figure 5(D). It can be seen from the magnification of the releasing curve, during the first 1 hour, NMS/CMS sample released faster than the pure NMS, and the release rate increased with the increasing proportion of the carrier. However, as indicated by the accumulative release percentage, 2:1 NMS/CMS, 1:1 NMS/CMS and 1:2 NMS/CMS exhibited dissolution patterns similar to the pure NMS. They all released about 95% of CMS within 24 h (93.1% for pure NMS, 96.1% for 2:1 NMS/CMS, 94.1% for 1:1 NMS/CMS and 93.7% for 1:2 NMS/CMS). Between them, 1:3 NMS/CMS showed improved release behavior in both rate and accumulative release percentage compared with pure NMS. These result demonstrated that, in the case of NMS/CMS, the improvement of dissolution rate can be attributed to the limited particle size and the conversion of crystalline state of NMS into amorphous phase but not the present of CMS. And the dissolution profile of drug can be regulated and controlled by mixing with different ratio of carrier.

### 3.6. Biodistribution of DZP

The concentration of DZP in blood and organs was measured by HPLC method at 45 min and 90 min after oral medication. As compared with 1:3 DZP/CMS, the DZP biodistribution after treatment of 1:3 DZP/MCM41 was also investigated due to the fact that MCM41 was a kind of mesoporous silica carrier widely used in drug sustained and controlled release area. In the present study, MCM41 with average particle size of 250 nm was chosen to eliminate the effect of size. Moreover, the distribution of pure DZP after oral administration was also determined and regarded as a reference.

**Figure 6.**
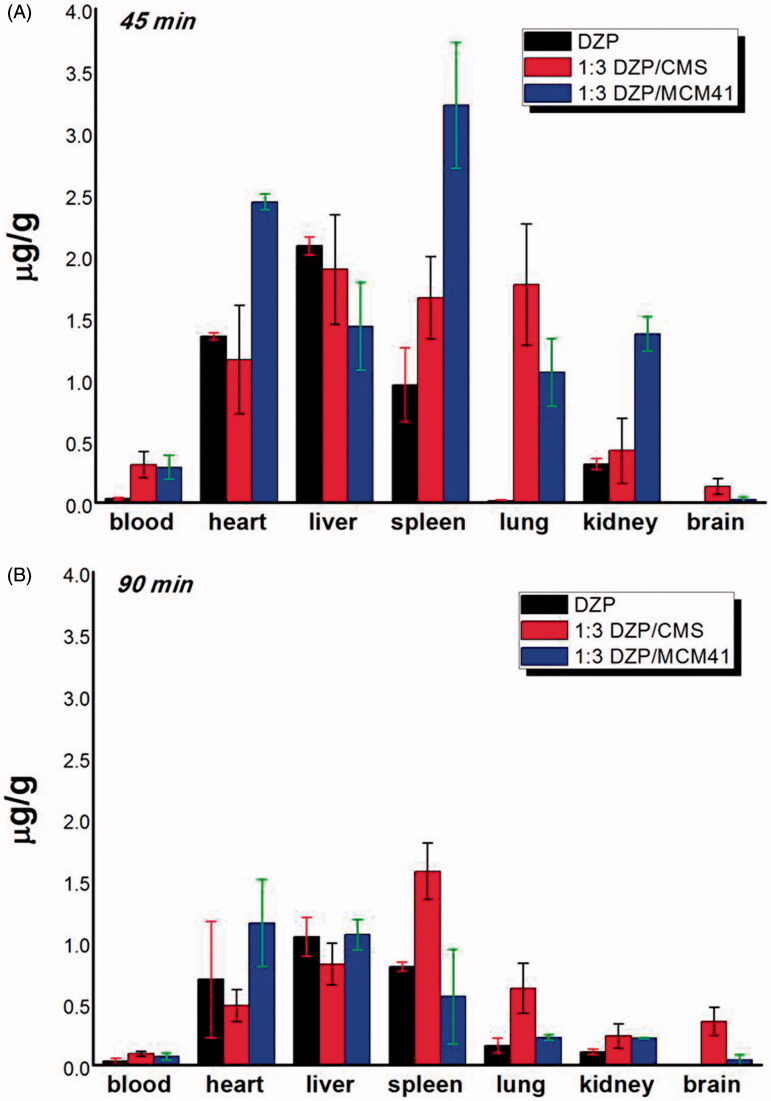
DZP contents in blood, heart, liver, lung, spleen and kidney at (A) 45 min; (B) 90 min postadministration.

As shown in Figure 6, at 45 min postadministration, DZP distributed themselves spatially in different tissues, and remarked difference can be observed between the three groups. In detail, 1:3 DZP/CMS sample had a higher drug distribution in blood, lung and brain and lower drug content in heart, whereas 1:3 DZP/MCM41 sample had a higher distribution in heart, spleen and kidney compared with their counterparts. The drug plasma concentrations of 1:3 DZP/CMS sample and 1:3 DZP/MCM41 sample were 8.57-fold and 7.94-fold higher than pure DZP sample, respectively. In lung, the content of DZP from 1:3 DZP/CMS sample was 124.94-fold and 1.67-fold higher than that of pure DZP and 1:3 DZP/MCM41 sample, respectively. Besides, the distribution of drug in brain from 1:3 DZP/CMS sample was 19.55-fold of that from DZP sample and 5.03-fold of that from 1:3 DZP/MCM41 sample. Meanwhile, in heart and spleen, the drug content of 1:3 DZP/MCM41 sample was 1.81-fold and 3.37-fold of that of DZP sample, and 2.11-fold and 1.96-fold of that from 1:3 DZP/CMS sample, respectively. It seemed that, after administration, 1:3 DZP/CMS sample and 1:3 DZP/MCM41 sample showed higher bioavailability in blood and organs as compared to pure DZP.

At 90 min postadministration, all three samples had a comparable decrease of drug content in blood, heart and live. Compared to the drug concentration at 45 min postadministration, all samples had 48%–58% decrease in heart and 26%–56% decrease in liver, respectively. In kidney and spleen, the DZP content of 1:3 DZP/MCM41 sample suffered greatly attenuation and decreased to a lower level (about 17% and 15% of the drug content at 45 min postadministration, respectively). Meanwhile, the plasma concentration of DZP from 1:3 DZP/CMS sample was 2.71-fold and 1.30-fold of that from pure DZP sample and 1:3 DZP/MCM41 sample, respectively. In heart and lung, the drug content of 1:3 DZP/MCM41 sample and 1:3 DZP/CMS sample remained the highest, respectively. It is worth mentioning that, in brain, the content of DZP from 1:3 DZP/CMS sample was 2.71-fold higher than that at 45 min postadministration and was 62.31-fold and 7.72-fold of that from pure DZP sample and 1:3 DZP/MCM41 sample, respectively. This may contribute to the treatment of anxiety and seizures. Moreover, at 90 min postadministration, remarkable distribution of DZP in spleen was emerged from 1:3 DZP/CMS sample, which remained almost the same drug content as at 45 min postadministration, and was 2.85-fold higher than the 1:3 DZP/MCM41 sample and 1.96-fold higher than the pure DZP sample. The drug accumulated in spleen may play the role of drug reservoirs to remain a higher DZP concentration. From these results, it was speculated that DZP/CMS drug loading system had altered the DZP biodistribution in mice and had the tendency of brain targeting and lung targeting. Therefore, it is reasonable to presume that the change of DZP biodistribution could be attributed to the presence of CMS, but not the passive targeting effect of carriers or the decrease of DZP particle size. However, the targeting mechanisms of CMS should be further studied.

### 3.7. Pharmacodynamic study of NMS

**Figure 7.**
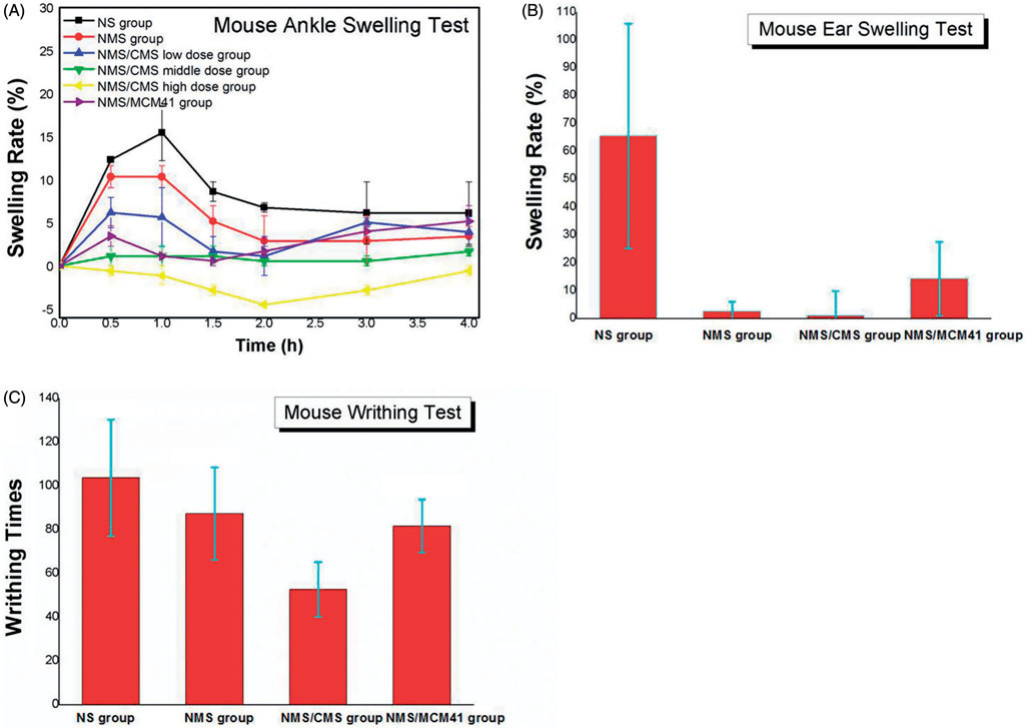
Anti-inflammatory analgesic effects of samples. (A) The anti-inflammatory effects of NMS formulations on mouse ankle swelling induced by carrageenan; (B) the effects on mouse ear swelling caused by xylene; (C) the analgesic effects of samples on acetic acid-induced writhing in mice.

Anti-inflammatory effects of NMS, 1:3 NMS/CMS and 1:3 NMS/MCM41 were systematically evaluated and compared by mouse ankle swelling test, mouse ear swelling test and mouse writhing test, and the results were shown in Figure 7 and Table 2, respectively. It can be seen that in Table 2, both 1:3 NMS/CMS and 1:3 NMS/MCM41 can obviously inhibit the ankle swelling, ear swelling and the acetic acid-induced writhing in rats in comparison with pure NMS. Among them, the anti-inflammatory effect of 1:3 NMS/CMS significantly outperformed that of NMS and 1:3 NMS/MCM41.

**Table 2.** Inhibition rate (%) of NMS-CMS 3:1 and NMS-MCM41 3:1 in MAST, MEST and MWT.

Inhibition rate (%)	MAST							
Sample	0.5 h	1 h	1.5 h	2 h	3 h	4 h	MEST	MWT
1:3 NMS/CMS	904.3 ± 101.1	328.7 ± 29.1	244.2 ± 40.4	167.3 ± 16.2	180.0 ± 20.0	175.0 ± 25.5	102.2 ± 14.5	312.1 ± 62.0
1:3 NMS-MCM41	698.1 ± 97.0	318.9 ± 2.6	260.1 ± 20.8	133.2 ± 16.7	59.7 ± 19.9	58.2 ± 38.8	82.3 ± 21.7	134.5 ± 60.9

#### 3.7.1. Mouse ankle swelling test

Ankle swelling responses in each group at 0.5–4 h after injection were presented in Figure 7(A). In NS group (negative control), maximum swelling (15.5%) was observed at 1 h, and swelling gradually diminished thereafter. However, swelling curves in other groups peaked at 0.5 h, and swelling began to decrease spontaneously. Both NMS/CMS and NMS/MCM41 can obviously inhibit the ankle swelling degree compared to the pure NMS. Under the same dose, NMS/CMS (NMS/CMS middle-dose group, which maximum swelling rate was less than 1.5%) had a stronger effect than NMS/MCM41. It should be noticed that 1:3 NMS/CMS dose dependently and significantly inhibited the swelling induced by carrageenan, and almost no swelling was observed in NMS/CMS middle-dose group and the NMS/CMS high-dose group and indicated the activity of NMS/CMS in swelling inhibition.

#### 3.7.2. Mouse ear swelling test

Mouse ear swelling test was carried out to further evaluate the anti-inflammatory potential of 1:3 NMS/CMS and 1:3 NMS/MCM41, and the results were shown in Figure 7(B) and Table 2, respectively. In the NS group, xylene caused significant increase in ear weight, and the swelling rate was as high as 67%. By application of NMS, a lower increase in ear weight was observed, the swelling rate was 32.4%, and this value was used as positive control. As can be seen from Figure 7(B), CMS as well as MCM41 contributed to the inhibition of swelling, and the inhibition rate was as high as 102.2% and 82.3%, respectively. The results demonstrated the enhancement of swelling inhibition activity of NMS by incorporating into CMS or MCM41. It seemed that NMS/CMS was found to be more powerful in suppressing ear swell in mice compared to NMS/MCM41.

#### 3.7.3. Mouse writhing test

The antinociceptive effects of NMS/CMS, NMS/MCM4 and pure NMS were estimated by acetic acid-induced mouse writhing test. Figure 7(C) and Table 2 showed the writhing responses in mice, respectively. It can be seen that the number of writhes induced by acetic acid was significantly reduced by administration of 1:3 NMS/CMS due to the improvement of pain threshold in mice, and the inhibition rate was as high as 312.1%. 1:3 NMS/MCM41 had a weaker antinociceptive effect than NMS/CMS, the inhibition rate was 134.5% which was also stronger than that of pure NMS, and the average number of writhes was reduced from 103.7 (negative control) to 81.7.

The results demonstrated that after loading into CMS, the swelling inhibition effect and analgesic activity of NMS were significantly enhanced. Moreover, NMS/CMS showed dose-related inhibitory effect on ankle swelling of mouse.

## 4. Conclusions

In summary, proline-derivative templated mesoporous silica spheres with a large number of curved channels were biomimetically synthesized in water solvent using CSDA method and were successfully developed as an effective nanocarrier to improve the drug release behavior and the bioavailability of poorly water-soluble drugs. During this process, the curved mesostructural was induced by the dynamic behavior of *N*-palmitoyl-l-proline and imprinted along with the silica deposition procedure. Drugs can be incorporated into CMS with high efficiency and drugs transformed from crystalline state to amorphous state due to the space confinement. As a result, the dissolution rates of the drugs were significantly improved. The biodistribution study suggested that, after loaded into CMS, the DZP distribution in mice had been altered and showed the tendency of brain targeting and lung targeting. In particular, we will focus on the targeting mechanisms of CMS *in vivo* in the next step. Besides, the pharmacodynamic experiment demonstrated that the anti-inflammation effect and analgesia action of NMS were obviously improved by incorporating into CMS, which undoubtedly favored the clinical treatment.

We believe that these findings will provide useful information on biological application of CMS and are of great importance for controlled drug delivery and release system of nanoporous silica-based drug carriers.
